# PFE-Det: Progressive Feature Evolution for Small Object Detection in UAV Aerial Images

**DOI:** 10.3390/s26155003

**Published:** 2026-08-06

**Authors:** Aolin Fang, Yongzi Zhang, Xiaotong Dong, Liuyang Gu, Shengshi Li, Daoheng Zhu, Xiuchun Xiao

**Affiliations:** 1 College of Mathematics and Computer Science, Guangdong Ocean University, Zhanjiang 524088, China; aolinf777@163.com (A.F.); 13699876922@stu.gdou.edu.cn (Y.Z.); 2 College of Electronics and Information Engineering, Guangdong Ocean University, Zhanjiang 524088, China; dongxiaotong@stu.gdou.edu.cn (X.D.); guliuyang@stu.gdou.edu.cn (L.G.); lishengshi@gdou.edu.cn (S.L.)

**Keywords:** UAV object detection, small object detection, progressive feature evolution, state space model, multi-scale feature fusion, receptive field modeling, gated nonlinear selection, aerial image analysis

## Abstract

Object detection in UAV aerial images remains fundamentally constrained by extremely small object scales, strong background interference, and progressive structural information degradation along the feature extraction pipeline. Current small-object detection methods suffer from two fundamental deficiencies rooted in their convolutional feature extraction pipelines: the smoothing effect of strided convolutions in early layers, which attenuates fine-grained details before backbone processing, and the feature overwriting phenomenon, where sequential transformations progressively erase structural information from earlier layers. We propose PFE-Det (Progressive Feature Evolution Detector), built upon the DEIM framework and constructing a continuous optimization pathway across three stages. A Feature Adaptive Enhancement Network (FAENet) is adopted as a front-end preprocessor to decouple high- and low-frequency components via a Laplacian pyramid at the input stage, mitigating early-layer smoothing at the input. A Multi-Receptive-Field Adaptive Fusion Module (MFAM) is designed to reorganize single-path features into structure-retaining and progressive enhancement paths and is further coupled with hierarchical receptive-field modeling, suppressing feature overwriting through multi-scale context modeling. A Multi-Path Gated State Space Modeling Block (MG-SSM Block) couples HSM-SSD-based long-range dependency extraction with Convolutional Gated Linear Units (CGLU) for adaptive feature selection in the encoder. Experiments on VisDrone2019 demonstrate an AP of 0.225 and an ${\mathrm{AP}}_{s}$ of 0.134, yielding a 13.5% relative improvement in small-object precision over the baseline. Cross-dataset evaluations on DIOR and UAVVaste, each under independent training and testing, further support the effectiveness of progressive feature evolution for UAV small-object detection.

## 1. Introduction

Unmanned Aerial Vehicles (UAVs), owing to their flexible maneuverability, rapid deployability, and low operational cost, have been widely adopted in civilian domains such as traffic surveillance, agricultural management, and disaster response [1]. As intelligent perception demands grow in these scenarios, object detection has become the foundational task of UAV vision systems, directly determining their overall performance [2]. However, UAV aerial images fundamentally differ from ground-level camera captures in their visual characteristics: constrained by flight altitude and wide-angle optics, objects in such images typically appear with extremely small pixel footprints, are densely distributed across texturally complex backgrounds, and exhibit drastic scale variations with changes in altitude [2,3]. Taking the widely used VisDrone2019 benchmark [2] as a representative example, small-object instances (normalized area a<0.01) account for approximately 97–98% of all annotations [4]. The dense object distribution and intense background interference together constitute extremely harsh detection conditions, driving UAV aerial small-object detection to emerge as a critical research frontier in computer vision [3,5].

These characteristics make reliable small-object perception indispensable for practical UAV intelligence. Missed or fragmented detections of distant vehicles, pedestrians, or debris can directly degrade traffic-monitoring accuracy, agricultural inspection completeness, and post-disaster search effectiveness. Consequently, preserving fine-grained structural cues—rather than attempting to recover them only after severe early degradation—is a prerequisite for deploying detectors under the dense, scale-varying, and clutter-dominated conditions typical of aerial sensing.

Over the past decade, deep learning has driven successive paradigm shifts in object detection—from two-stage detectors (Faster R-CNN [6]), through multi-scale feature pyramids (FPN [7]) and single-stage real-time frameworks (the YOLO series, including the UAV-oriented TPH-YOLOv5 [8]), to end-to-end Transformer detectors. Among the latter, RT-DETR [9] first achieved real-time end-to-end detection, D-FINE [10] refined localization through fine-grained distribution modeling, and DEIM [11] accelerated convergence via dense one-to-one matching with 50% training time reduction, collectively representing the current real-time detection frontier. However, despite notable achievements on general benchmarks, these methods still exhibit systematic deficiencies when applied to UAV aerial small-object detection, rooted in two long-neglected fundamental defects in convolutional network architectures [5,12,13].

The first is the smoothing effect inherent in early convolutional layers. Strided convolutions and pooling layers, standard designs in the vast majority of convolutional networks, inevitably cause systematic loss of high-frequency detail information during spatial downsampling [14]. This mechanism has negligible impact in general scenarios, but in low-resolution small-object settings, its attenuation of fine-grained edge and structural information is disproportionately amplified, causing extremely small objects with sparse pixel representations to suffer irreversible feature degradation before even entering the backbone network [5,14]. The second is the feature overwriting phenomenon prevalent in deep feature propagation. As features are updated layer by layer along a single path, the continuous transformations applied by successive layers persistently overwrite the feature representations from earlier layers, causing the physical structural information contained in early layers to be progressively erased as network depth increases [12]. Information bottleneck theory indicates that, as the number of forward-propagation layers accumulates, the mutual information loss between the network input and the target task prediction gradually accumulates, and fine-grained structures progressively vanish in deep-layer features, severely undermining the semantic discriminability of small objects amid dense background interference [5,12,13]. These two fundamental defects compound each other in a cascading manner: the smoothing effect degrades fine-grained features at the entry point, while feature overwriting prevents recovery of this lost information in subsequent layers, collectively constraining further breakthroughs in UAV aerial small-object detection. Nevertheless, most existing methods still lack systematic solutions that target both defects. This work is motivated by a concrete gap: although early-layer smoothing and deep-layer feature overwriting are known to cascade along the convolutional feature pathway and are particularly damaging to UAV small objects, most existing remedies either compensate at the detection head or enhance an isolated module, without constructing an ordered, stage-wise optimization path that jointly mitigates both defects from input to encoder. This gap directly motivates the progressive feature evolution framework developed in thiswork.

To mitigate these limitations, researchers have explored potential improvement paths from multiple technical dimensions, each addressing different aspects of the two defects. For the feature overwriting problem, global dependency modeling offers an alternative to single-path sequential propagation: selective state space models (SSM) represented by Mamba [15] achieve powerful long-range dependency capture with linear time complexity; VMamba [16] further migrated the SSM mechanism to general visual tasks through 2D selective scanning; EfficientViM [17] substantially reduced SSM inference overhead via the Hidden State Mixer-based State Space Duality (HSM-SSD) mechanism. For the smoothing effect and the associated loss of multi-scale structural context, lightweight multi-receptive-field modeling offers a complementary solution: LWGANet [18] achieves coordinated multi-scale spatial information extraction from point-level details to global semantics through channel grouping and scale-specific paths. At the architectural level, MetaFormer [19] demonstrated that the general framework of “normalization–Token Mixer–residual fusion” is the key driver of competitive performance in vision models. However, how to organically integrate these technical lines into a unified detection framework, constructing a coherent and systematic optimization path specifically targeting the two defects of smoothing effect and feature overwriting, remains largely unexplored, leaving a critical gap between isolated technical advances and the systematic detection performance demanded by real-world UAV applications.

To address these challenges, this paper proposes PFE-Det (Progressive Feature Evolution Detector), a UAV aerial small-object detection model based on progressive feature evolution. This work organizes frequency decoupling, multi-receptive-field modeling, and state-space interaction into an ordered, stage-wise feature evolution pathway tailored to UAV small-object detection. The model takes DEIM [11] (D-FINE-S [10] configuration) as the detection baseline and constructs a continuous optimization path of “structural enhancement—multi-scale reconstruction—global dependency modeling and adaptive selection” around the evolution of feature representations from low-level to high-level. At the input stage, FAENet [20] is adopted as a front-end preprocessor to explicitly decouple high- and low-frequency components of the input image via a Laplacian pyramid, using a low-frequency structure-guided affine transformation mechanism to restore high-frequency details layer by layer, mitigating the smoothing effect of early convolutions from the source. At the backbone stage, MFAM is designed to reorganize the single feature path into structure-retaining and progressive enhancement paths, and is further coupled with a hierarchical receptive-field unit adapted from LWGA [18], covering point-level to global spatial contexts. This dual-level design effectively prevents fine-grained structural information from being overwritten during continuous feature transformations. At the encoder stage, the MG-SSM Block couples HSM-SSD-based long-range dependency extraction [17] with the adaptive nonlinear feature selection of Convolutional Gated Linear Units (CGLU) within a unified residual framework. This work conducts experiments primarily on the VisDrone2019 dataset [2,4] and performs cross-dataset evaluation on the DIOR [21] and UAVVaste [22] datasets. Experimental results demonstrate that PFE-Det achieves an overall AP of 0.225 and a small-object precision ${\mathrm{AP}}_{s}$ of 0.134, yielding a 13.5% relative improvement over the baseline.

The main contributions of this paper are summarized as follows:

1. We formulate progressive feature evolution as a unified detection framework that constructs an ordered optimization pathway—structural enhancement, multi-scale reconstruction, and global dependency modeling with adaptive selection—specifically targeting the cascading defects of early-layer smoothing and deep-layer feature overwriting in UAV small-object detection. In contrast to recent DEIM-based detectors that primarily strengthen one-to-one matching and training efficiency [11], RT-DETR-style detectors that focus on hybrid encoding and query selection [9], and Mamba-/SSM-based detectors that emphasize global scanning or linear-complexity sequence modeling [15,16,17,23], the proposed framework applies stage-wise interventions along the full feature extraction pipeline and empirically exhibits an ordered inter-module dependency along this pathway.
2. We design MFAM with a dual-level architecture that couples an outer multi-path decomposition layer (structure-retaining versus progressive enhancement) with an inner hierarchical receptive-field unit adapted from LWGA [18]. This coupling enables fine-grained structural information to be retained and propagated under deep backbone transformations, mitigating the feature-overwriting risk that remains when multi-receptive-field modeling is embedded in a conventional single-path backbone.
3. We construct the MG-SSM Block that couples HSM-SSD-based long-range dependency modeling [17] with spatially aware CGLU-based gated feature selection within a unified residual framework, extending the linear-complexity SSM paradigm from pure dependency propagation to a co-driven “dependency extraction–adaptive selection” mechanism for dense aerial detection.
4. We adopt FAENet [20] as a lightweight front-end frequency-decoupling preprocessor (only 0.04 M additional parameters) to mitigate early smoothing at the source. Ablation studies further show that its benefit is most evident within the complete progressive pathway. Experiments on VisDrone2019, together with independent evaluations on DIOR and UAVVaste, demonstrate a 13.5% relative ${\mathrm{AP}}_{s}$ improvement over the DEIM baseline and support the cross-dataset applicability of the proposed framework in aerial and remote sensing scenarios.

## 2. Related Work

### 2.1. Object Detection Paradigm Evolution and Real-Time Detection Transformers

Deep learning object detection has progressed from two-stage detectors such as Faster R-CNN [6] to single-stage real-time frameworks and end-to-end Transformer paradigms. DETR [24] introduced set prediction via bipartite matching, while subsequent work improved real-time efficiency and localization: RT-DETR [9] through a hybrid encoder and IoU-aware query selection, D-FINE [10] through fine-grained distribution refinement, and DEIM [11] through dense one-to-one matching. YOLOv9 [12] further highlighted information loss during deep feature propagation via Programmable Gradient Information (PGI), motivating structural-information protection in the present study. We adopt DEIM (D-FINE-S) as the detection baseline for its balance of accuracy, real-time performance, and training efficiency.

Beyond matching and training-efficiency advances, recent DETR-based detectors have also rethought the multi-scale feature hierarchy to alleviate multi-scale modeling limitations and weak small-object cues under quadratic-attention constraints. Liu et al. [25] develop F-DETR with a heterogeneous-scale multi-branch structure that integrates multi-scale features into the DETR decoder, promoting local–global interaction while controlling sequence length. In a complementary direction, DETR++ [26] aggregates multi-level backbone features with a Bidirectional Feature Pyramid before the Transformer encoder, showing that enriched multi-scale representations can improve DETR accuracy, particularly for small objects. Such multi-scale representation learning therefore expands the feature hierarchy available to set-prediction heads. Complementary to these detector-internal hierarchy designs, the present work instead reconstructs the upstream preprocessing–backbone–encoder pathway on a DEIM baseline so that structural information is preserved before decoding.

### 2.2. Small Object Detection for UAV Scenarios

UAV aerial object detection faces inherent challenges of extremely small object pixels, strong background interference, and drastic scale variation. The VisDrone dataset [2], covering 14 cities with over 2.6 million annotations, provides the most stringent benchmark in this domain. Recent surveys [3,5] have systematically revealed that existing solutions still suffer from fundamental deficiencies in protecting fine-grained structural information under dense backgrounds.

At the method level, existing approaches address different aspects but share a common limitation. TPH-YOLOv5 [8] introduced Transformer prediction heads for UAV detection but confined structural compensation to the detection stage without resolving feature degradation in the extraction process. CEASC [27] optimized sparse convolution in detection heads for efficiency but does not fully support complete structural modeling in dense scenes. RemDet [28] replaced the standard FFN with GatedFFN and reached the VisDrone performance frontier at 110 FPS, but its gated enhancement lacks co-design with global dependency modeling. Sunkara et al. [14] demonstrated the systematic fine-grained information loss caused by strided convolutions, a defect amplified in low-resolution small-object settings. Recent studies have further explored RT-DETR-based multi-scale attention mechanisms [29] and lightweight dynamic feature fusion architectures [30] for UAV small-object detection on VisDrone, but these approaches similarly lack systematic structural information protection throughout the feature extraction process. Overall, existing methods either focus on detection-stage compensation or lightweight efficiency optimization; a unified framework that systematically protects structural information throughout the entire feature extraction pipeline remains an open problem—one that this work directlyaddresses.

### 2.3. Multi-Scale Feature Fusion, Receptive Field Modeling, and Attention Mechanisms

Effective multi-scale feature fusion and broad receptive field coverage are core requirements for small object detection. FPN [7] established the foundational multi-scale paradigm through a top-down pathway with lateral connections; however, its single-path transfer mechanism continuously overwrites prior feature representations, causing low-level structural information to progressively weaken—this “feature overwriting” defect directly motivates the design of MFAM. For receptive field expansion, atrous convolution [31] and large-kernel convolution [32] have demonstrated the importance of multi-scale context aggregation. In lightweight multi-receptive-field modeling, LWGANet [18] divides channels into heterogeneous groups routed to scale-specific paths from point-level details to global semantics, achieving coordinated multi-scale extraction without additional computational overhead. Despite its excellent accuracy–efficiency tradeoff, LWGA in a single-path backbone still faces the risk of feature overwriting in UAV dense small-object scenarios. Beyond UAV detection, recent perception systems likewise emphasize hierarchical feature integration under complex backgrounds: BANet [33] introduces bidirectional feature aggregation and adaptive multi-scene perception for lane detection, illustrating that cross-scale complementary aggregation and scene-adaptive cue enhancement remain active design principles. In a related direction, CCNet [34] proposes a cross-modal co-attention network for light field salient object detection, assigning importance weights to focal slices and combining cross-complementary refinement with cross-modal hierarchical co-attention to fuse RGB and focal-stack features under layered interaction, followed by RGB-guided decoding. Such hierarchical complementary aggregation is conceptually related to the multi-path structural retention and gated selection pursued by MFAM and the MG-SSM Block; however, BANet targets lane geometry and CCNet targets light field saliency rather than dense aerial bounding-box detection, and neither addresses early-layer smoothing along a progressive UAV detection pathway.

In adaptive feature selection, SE Networks [35] and CBAM [36] model inter-channel and spatial dependencies for adaptive feature refinement, but lack fine-grained structural guidance. MetaFormer [19] revealed that the general “normalization–Token Mixer–residual fusion” framework, rather than specific Token Mixer implementations, is the key driver of competitive performance, providing the architectural backing for flexibly embedding different feature interaction mechanisms within a unified residual framework—a principle directly adopted in the MG-SSM Block design. Overall, existing multi-scale methods struggle to balance structural information protection and context modeling in single-patharchitectures.

### 2.4. Visual State Space Models and Gated Nonlinear Feature Modeling

The quadratic complexity of Transformer self-attention creates computational bottlenecks in high-resolution dense prediction, driving researchers to seek linear-complexity alternatives. Mamba [15] first demonstrated efficient global dependency modeling via the selective state space (S6) mechanism with approximately 5× inference throughput improvement over same-scale Transformers. VMamba [16] extended this mechanism to 2D vision tasks through four-path selective scanning and has been further adopted for UAV small-object detection with high-resolution feature pyramids [23]. Vision Mamba [37] further validated that visual models need not rely on self-attention.

Building on these foundations, EfficientViM [17] redesigns the SSD layer based on Hidden State Mixer State Space Duality (HSM-SSD), substantially reducing computation overhead while achieving state-of-the-art speed–accuracy tradeoffs on ImageNet-1K. However, its FFN adopts a standard per-channel nonlinear activation form, lacking targeted discriminative enhancement for dense object detection. MiM-ISTD [38] validated the feasibility of SSM for small-object detection in remote sensing, while MetaFormer Baselines [39] confirmed the robustness of replacing Token Mixers with customized feature interactionmechanisms.

In gated nonlinear modeling, the Gated Linear Unit (GLU) [40] established the foundational paradigm of adaptive gating control through element-wise multiplication of a linear branch and a gate branch. However, standard GLU and its variants ignore explicit spatial structure modeling in visual features. RemDet’s GatedFFN [28] demonstrated the superiority of gated multiplication in UAV detection, but its gating operates independently of long-range dependency modeling. The key open challenge is how to couple SSM-driven dependency modeling with spatially aware gated feature selection within a unified framework, which constitutes the core motivation for the MG-SSM Block design.

## 3. Method

### 3.1. Overall Framework

**Figure 1:**
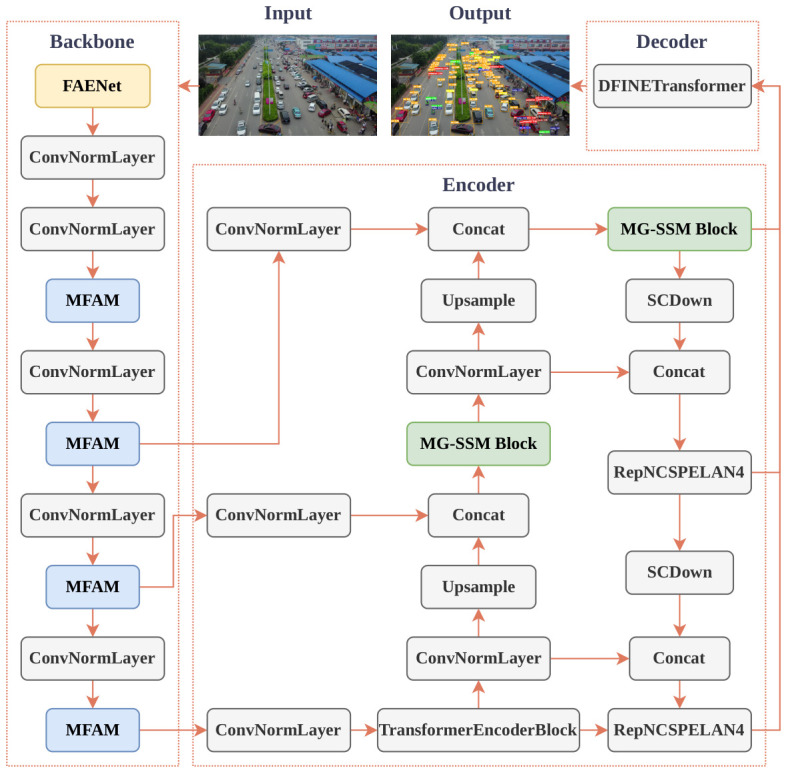
Overall architecture of PFE-Det. The three core modules (FAENet, MFAM, MG-SSM Block) are integrated into the backbone–encoder–decoder structure of DEIM, forming a continuous optimization path from structural enhancement to global dependency modeling. Module operators shown in the figure are defined as follows: ConvNormLayer is a convolution–normalization block; Upsample and Concat denote spatial upsampling and channel-wise concatenation; TransformerEncoderBlock is a multi-head self-attention encoder block; SCDown is a lightweight downsampling operator (pointwise convolution followed by depth-wise strided convolution); RepNCSPELAN4 is a multi-branch CSP-ELAN-style fusion block for cross-scale aggregation; and DFINETransformer is the D-FINE detection transformer decoder. Among them, ConvNormLayer, TransformerEncoderBlock, SCDown, RepNCSPELAN4, and DFINETransformer are inherited from the DEIM baseline.

As shown in Figure 1, this work performs a systematic reconstruction of the feature modeling process in the backbone–encoder–decoder structure of the DEIM (D-FINE) detection framework from the perspective of progressive representation optimization. Rather than simply stacking independent modules, the proposed method introduces level-specific modeling mechanisms that progressively optimize feature representations as they propagate from low-level to high-level.

Given an input image $I\in R^{H\times W\times 3}$, the overall feature flow can be expressed as

(1) $$I\to F_{\mathrm{pre}}\to F_{\mathrm{backbone}}\to F_{\mathrm{encoder}}\to F_{\mathrm{decoder}}\to \hat{Y},$$

where the four stages correspond to low-level structural enhancement, multi-scale spatial representation reconstruction, global dependency modeling with discriminative enhancement, and target prediction, respectively, and $\hat{Y}$ denotes the final detection output comprising predicted bounding boxes and category labels.

At the input stage, a Feature Adaptive Enhancement Network (FAENet) [20] preprocesses the raw image for structural enhancement. Although structurally integrated into the starting portion of the backbone, it functionally corresponds to the front-end feature enhancement stage ($F_{\mathrm{pre}}$). This stage employs high–low frequency decomposition. A structure-guided mechanism then improves the stability and discriminability of low-level features, thereby providing a reliable foundation for subsequent modeling.

At the backbone stage, a Multi-Receptive-Field Adaptive Fusion Module (MFAM) is applied during the multi-level feature extraction process (P2–P5) to perform multi-path decomposition and hierarchical receptive field modeling. This design enables local detail information and large-scale context to be adaptively fused in a unified framework, thereby realizing the transition from low-level structural representations to multi-scale spatial representations.

At the encoder stage, a Multi-Path Gated State Space Modeling Block (MG-SSM Block) is constructed as the feature interaction mechanism on the fused multi-scale features, coupling state-space-driven cross-position long-range dependency extraction with the adaptive nonlinear feature selection of CGLU within a unified residual framework, thereby achieving the transition from multi-scale spatial modeling to global dependency modeling and discriminative enhancement. As illustrated in Figure 1, besides the proposed MG-SSM Block, the hybrid encoder/decoder retains several DEIM baseline operators that are named and defined in the figure caption, with TransformerEncoderBlock kept on the P5 branch.

At the decoding stage, the model retains the original DFINETransformer from DEIM with the D-FINE-S settings (hidden dimension 256, three decoder layers, and three multi-scale feature levels) for unified modeling and target prediction of multi-scale features from the encoder.

The entire network thus forms a continuous optimization path of “structural enhancement—multi-scale reconstruction—global dependency modeling and adaptive selection,” enabling feature representations to progressively evolve and synergistically improve at different levels.

Key architectural settings are constrained either by the defaults of adopted components or by stage-wise capacity allocation under UAV small-object constraints (high-resolution early features, severe scale variation, and limited compute budget). Exact values and task-oriented rationales are specified in the corresponding module subsections below.

For end-to-end learning, PFE-Det retains the DEIM dense one-to-one matching strategy and the DEIMCriterion composite objective without introducing an additional loss term. With Hungarian matching between predictions and ground-truth objects, the overall training objective is

(2) $$L=\lambda_{\mathrm{mal}}L_{\mathrm{mal}}+\lambda_{\mathrm{bbox}}L_{\mathrm{bbox}}+\lambda_{\mathrm{giou}}L_{\mathrm{giou}}+\lambda_{\mathrm{fgl}}L_{\mathrm{fgl}}+\lambda_{\mathrm{ddf}}L_{\mathrm{ddf}},$$

where $L_{\mathrm{mal}}$ is the matching-aware classification loss, $L_{\mathrm{bbox}}$ and $L_{\mathrm{giou}}$ are the $L_{1}$ and GIoU box-regression losses, and $L_{\mathrm{fgl}}$ and $L_{\mathrm{ddf}}$ are the fine-grained localization and decoupled distillation focal terms from D-FINE/DEIM. Following the DEIM training configuration used in our experiments, the loss weights are set to $\lambda_{\mathrm{mal}}=1$, $\lambda_{\mathrm{bbox}}=5$, $\lambda_{\mathrm{giou}}=2$, $\lambda_{\mathrm{fgl}}=0.15$, and $\lambda_{\mathrm{ddf}}=1.5$ [10,11].

### 3.2. Front-End Preprocessing Based on Feature Adaptive Enhancement

**Figure 2:**
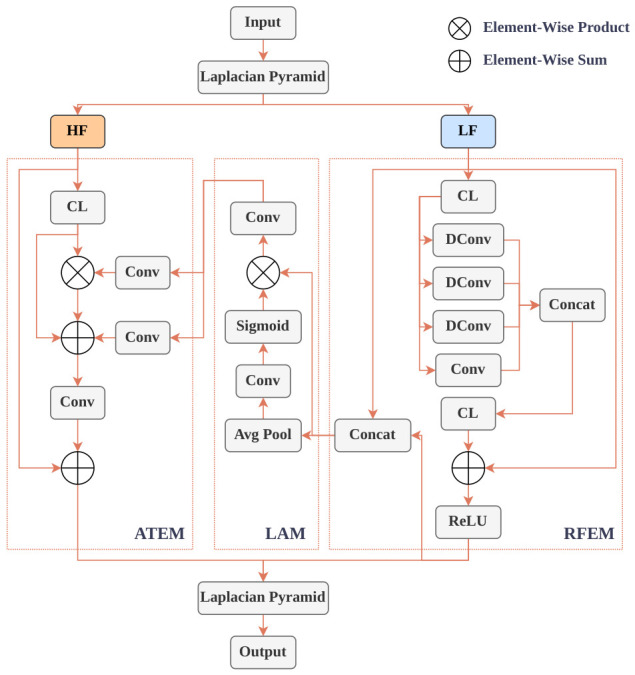
Architecture of the Feature Adaptive Enhancement Network (FAENet). CL denotes a convolutional layer block (composed of multiple convolution and activation layers); HF and LF denote the high-frequency and low-frequency components, respectively.

FAENet is adopted from prior work [20]. Accordingly, this subsection focuses on its role within the progressive pathway, while complete architectural details remain available in the original paper. In complex-scene object detection, feeding a raw image directly into the backbone may attenuate fine-grained details through early convolutional smoothing. FAENet is therefore placed as a front-end preprocessor that explicitly decouples high- and low-frequency components before any backbone convolution. Within the proposed framework, it provides structurally enhanced inputs to MFAM and the MG-SSM Block; the ablation study in Section 4.3 quantifies this dependency. Figure 2 illustrates its overallstructure.

FAENet constructs a Laplacian-pyramid decomposition of the input image I. Following the default configuration of the adopted FAENet implementation [20], and consistent with the need to recover source-resolution cues for extremely small UAV targets before backbone downsampling, we set the number of high-frequency pyramid levels to L=1, yielding one high-frequency residual ${\mathrm{HF}}_{1}$ and a lowest-resolution low-frequency structure LF:

(3) $$\{{\mathrm{HF}}_{1},\mathrm{LF}\}.$$

This single-level setting emphasizes native-resolution detail recovery while keeping the front-end overhead small (only 0.04 M additional parameters); deeper pyramids would add multi-scale residuals at the cost of extra upsampling/fusion computation before any detection benefit is guaranteed. The low-frequency component is enhanced by a Receptive Field Fusion Enhancement Module (RFEM) with multi-branch convolutions of different receptive fields, while a Lightweight Attention Module (LAM) produces guidance features that condition subsequent detail recovery. The high-frequency component is then corrected by an Affine Transformation Enhancement Module (ATEM) under structure-guided modulation:

(4) $${\mathrm{HF}}_{1}^{\prime }={\mathrm{HF}}_{1}+{\mathrm{HF}}_{1}⊙S\left(M_{1}\right)+B\left(M_{1}\right),$$

where $M_{1}$ denotes the upsampled guidance from the low-frequency pathway, and S(·) and B(·) generate scale and bias terms. The enhanced components are finally reconstructed by progressive upsampling and summation,

(5) $$I^{\prime }=P_{\mathrm{recon}}\left({\mathrm{LF}}^{\prime },\left\{{\mathrm{HF}}_{1}^{\prime }\right\}\right),$$

producing an input representation with improved structural stability and detail integrity before backbone processing.

### 3.3. Multi-Receptive-Field Adaptive Fusion Module

**Figure 3:**
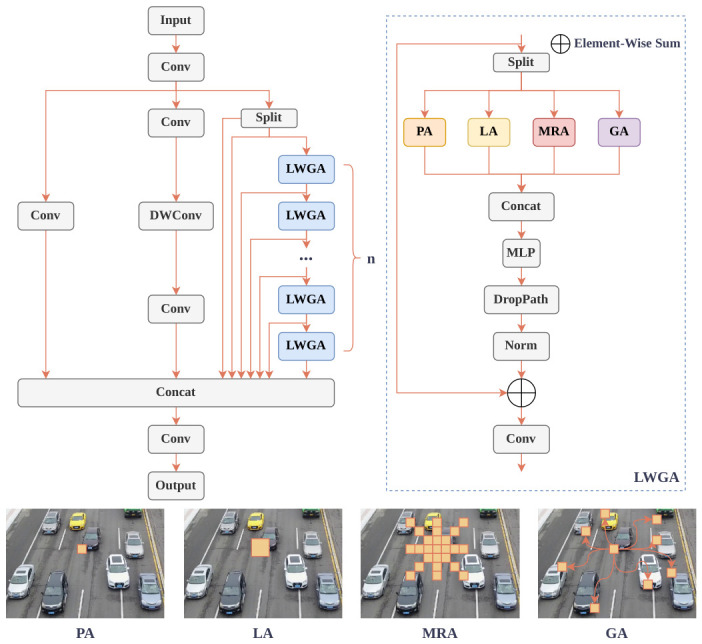
Overall architecture of the MFAM. The module adopts a dual-level design: the outer layer performs multi-branch feature decomposition and aggregation; the inner LWGA unit models hierarchical receptive fields from point-level to global contexts.

To overcome the feature overwriting defect inherent in single-path convolutional architectures, MFAM adopts a dual-level design: an outer multi-branch decomposition layer that separates structural preservation from progressive feature enhancement, and an inner LWGA unit that models spatial context hierarchically from point-level detail to global semantics. Together, these two levels enable fine-grained structural information to be retained and propagated stably across deep network layers. The overall architecture is shown in Figure 3.

#### 3.3.1. Multi-Branch Feature Decomposition and Reorganization

As discussed in Section 1, single-path convolutional architectures suffer from the persistent overwriting of early-layer structural information during sequential feature transformations.

To alleviate this, a multi-path feature decomposition mechanism is introduced to reorganize the single-path feature flow into multiple paths with different functional attributes, simultaneously achieving structural preservation and feature evolution within the same representation framework.

Specifically, given input feature $F\in R^{C\times H\times W}$, an intermediate representation is first obtained through channel mapping:

(6) Z=ϕ(F),

where ϕ(·) denotes the feature mapping function. Four structure-retaining paths $B=\{Z_{1}^{\left(b\right)},Z_{2}^{\left(b\right)},Z_{3}^{\left(b\right)},Z_{4}^{\left(b\right)}\}$ are then constructed from this intermediate feature: two complementary streams are produced by dual lightweight mappings (pointwise projection and depth-wise local reorganization), and two additional streams are obtained by splitting the intermediate feature along the channel axis. This four-stream outer topology is a constrained reorganization of MFAM that supplies complementary local structural views for later aggregation with the progressive pathway, matching the four-level receptive-field hierarchy introduced below. In parallel, a progressive enhancement pathway is constructed for gradual feature evolution and expression strengthening through *n* sequentially stacked transformation stages:

(7) $$Z_{1}^{\left(r\right)}=H_{1}\left(Z^{\left(b\right)}\right),\;Z_{i}^{\left(r\right)}=H_{i}\left(Z_{i-1}^{\left(r\right)}\right),\;i=2,\ldots ,n,$$

where $H_{i}\left(\cdot \right)$ denotes the feature transformation function of the *i*-th stage, forming the progressive enhancement sequence $E=\{Z_{1}^{\left(r\right)},Z_{2}^{\left(r\right)},\ldots ,Z_{n}^{\left(r\right)}\}$. Across backbone stages P2–P5, we set n=(1,2,2,2). Because P2 operates at the highest spatial resolution—where UAV small-object cues are richest but per-layer cost is also highest—a shallower progressive path (n=1) is used to preserve fine structures under a limited early-stage budget; from P3 onward, n=2 increases semantic capacity as resolution decreases and contextual discrimination becomes more critical.

This multi-path decomposition effectively decouples structural information retention from progressive feature evolution, preventing structural details from being repeatedly overwritten during continuous transformations.

#### 3.3.2. Hierarchical Receptive Field Modeling

After multi-path feature decomposition, each path’s features primarily retain local structural information without explicitly modeling contextual relationships across different spatial ranges. However, in remote sensing scenarios, object-scale variation is significant and backgrounds are complex, requiring feature representations to simultaneously cover multi-scale information from local details to global semantics.

To this end, a hierarchical receptive field modeling mechanism is introduced. Consistent with the channel grouping used in LWGA [18], the input feature $Z\in R^{C\times H\times W}$ is partitioned into K=4 sub-features along the channel dimension, corresponding to four receptive field levels: point-level, local neighborhood, medium-scale context, and globaldependence:

(8) $$Z=[Z_{1},Z_{2},Z_{3},Z_{4}].$$

We adopt K=4 as a constrained design choice aligned with LWGA’s equal channel budgets over these four ranges, which matches the drastic within-image scale variation typical of UAV scenes (from pixel-level targets to broader contextual structures). Correspondingly, LWGA operators indexed by stages 0–3 are applied on backbone stages P2–P5 so that the hierarchical receptive-field schedule remains aligned with the multi-resolution featurepyramid.

For each sub-feature $Z_{k}$, a transformation function $K_{k}\left(\cdot \right)$ with a different spatial modeling mechanism is applied:

(9) $$Z_{k}^{\prime }=K_{k}\left(Z_{k}\right),\;k=1,2,3,4.$$

Different $K_{k}\left(\cdot \right)$ form a hierarchy from small to large in terms of receptive field range, forming progressively increasing representational capability from local details to global semantics. After obtaining sub-features at each level, they are concatenated along the channel dimension and fused through a nonlinear mapping:

(10) $$Z_{\mathrm{cat}}=\mathrm{Concat}\left(Z_{1}^{\prime },Z_{2}^{\prime },Z_{3}^{\prime },Z_{4}^{\prime }\right),\;Z_{\mathrm{fuse}}=F\left(Z_{\mathrm{cat}}\right),$$

where F(·) denotes the feature fusion mapping composed of pointwise convolution, normalization, and nonlinear activation. A residual-style update mechanism is introduced to enhance stability:

(11) $$\hat{Z}=Z+W\left(Z_{\mathrm{fuse}}\right),\;Z^{\left(l\right)}={\mathrm{Conv}}_{1\times 1}\left(\hat{Z}\right).$$

#### 3.3.3. Multi-Path Feature Aggregation and Output Reconstruction

After multi-path feature decomposition and hierarchical receptive field modeling, all paths’ features are aggregated and reconstructed at the module output stage through concatenation and an output projection function:

(12) $$Y_{\mathrm{cat}}=\mathrm{Concat}\left(B,E\right),\;Z_{\mathrm{out}}=\Psi \left(Y_{\mathrm{cat}}\right),$$

where Ψ(·) denotes the output projection function implemented by a pointwise convolution for cross-path information integration and channel reconstruction.

### 3.4. Multi-Path Gated State Space Modeling Block

**Figure 4:**
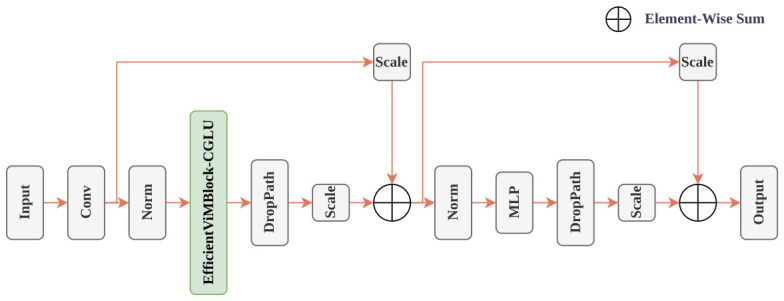
Overall architecture of the MG-SSM Block.

The MG-SSM Block couples state-space-driven long-range dependency extraction with a Convolutional Gated Linear Unit (CGLU) for adaptive nonlinear feature selection within a unified two-stage residual framework. This design enables the encoder to capture global cross-position dependencies at linear complexity while performing targeted feature selection, extending the purely linear SSM paradigm into a co-driven modeling approach. In the hybrid encoder, MG-SSM Blocks are placed on the P3 and P4 top-down fusion branches: these mid-level maps jointly retain sufficient spatial detail for small objects and enough semantic context for long-range aggregation under complex aerial backgrounds, while avoiding the quadratic attention cost of applying heavy global mixing at the highest-resolution maps. The coarsest P5 branch retains the baseline TransformerEncoderBlock to preserve strong semantic encoding at the lowest resolution. Following the default feed-forward expansion of EfficientViM [17], the CGLU branch uses mlp_ratio=4.0 as a constrained capacity setting for gated nonlinear selection. The block architecture is shown in Figure 4.

#### 3.4.1. Feature Mapping and Overall Structure

Given input feature $X\in R^{C\times H\times W}$, a channel alignment is first performed through linear mapping:

(13) $$X_{0}={\mathrm{Conv}}_{1\times 1}\left(X\right).$$

The MG-SSM Block adopts a two-stage transformation structure. In the first stage, a feature interaction mechanism is introduced after normalization:

(14) $$X_{1}=D_{1}\left(X_{0}\right)+S_{1}\left(F_{\mathrm{mix}}\left(N_{1}\left(X_{0}\right)\right)\right),$$

where $N_{1}\left(\cdot \right)$ denotes a LayerNorm operator applied to the first-stage input $X_{0}$, $D_{1}\left(\cdot \right)$ denotes the residual trunk mapping, $S_{1}\left(\cdot \right)$ denotes a stabilizing mapping with residual scaling and path modulation, and $F_{\mathrm{mix}}\left(\cdot \right)$ denotes the feature mixing mapping that integrates local convolutional modeling with sequence-rearrangement-based interaction mechanisms, coupling state space modeling and gated nonlinear enhancement. In the second stage, a nonlinear mapping is applied for further expression enhancement:

(15) $$X_{2}=D_{2}\left(X_{1}\right)+S_{2}\left(G\left(N_{2}\left(X_{1}\right)\right)\right),$$

where $N_{2}\left(\cdot \right)$ denotes a LayerNorm operator of the same type as $N_{1}\left(\cdot \right)$ but with independent learnable affine parameters, applied to the second-stage input $X_{1}$, and G(·) denotes the feature enhancement mapping composed of per-channel nonlinear feed-forward transformation. Therefore, $N_{1}$ and $N_{2}$ share the same normalization form; they differ in the stage at which they act and in their independently parameterized affine transforms. The overall mapping of MG-SSM Block is $M\left(X\right)=X_{2}$.

#### 3.4.2. State-Space-Driven Feature Interaction

In the first-stage feature mixing process, a state-space-driven feature interaction mechanism is introduced within $F_{\mathrm{mix}}\left(\cdot \right)$ to enhance the model’s ability to model long-range dependencies. Given normalized input feature $X_{0}^{\prime }=N_{1}\left(X_{0}\right)\in R^{C\times H\times W}$, a local depth-wise convolution is first applied for structural reorganization with per-channel path modulation:

(16) $$X^{\left(1\right)}=X_{0}^{\prime }+\alpha_{1}⊙\left(C_{1}\left(X_{0}^{\prime }\right)-X_{0}^{\prime }\right),$$

where $C_{1}\left(\cdot \right)$ denotes a depth-wise convolution mapping and $\alpha_{1}\in R^{C\times 1\times 1}$ is a learnable per-channel modulation parameter. The feature is then rearranged into sequence form $X_{s}^{\left(1\right)}\in R^{C\times L},L=H\times W$, and a state-space-driven interaction is applied:

(17) $$X^{\left(2\right)}=X^{\left(1\right)}+\alpha_{2}⊙\left(U_{\mathrm{sp}}\left(S_{\mathrm{ssm}}\left(X_{s}^{\left(1\right)}\right)\right)-X^{\left(1\right)}\right),$$

where $S_{\mathrm{ssm}}\left(\cdot \right)$ denotes the state space feature transformation based on the HSM-SSD module from EfficientViM [17], and $U_{\mathrm{sp}}\left(\cdot \right)$ denotes the rearrangement operation that restores the sequence representation to the 2D spatial structure. A local convolution refinement is further introduced:

(18) $$X^{\left(3\right)}=X^{\left(2\right)}+\alpha_{3}⊙\left(C_{2}\left(X^{\left(2\right)}\right)-X^{\left(2\right)}\right),$$

Analogous to $\alpha_{1}$, the coefficients $\alpha_{2},\alpha_{3}\in R^{C\times 1\times 1}$ are learnable per-channel modulation parameters (implemented with sigmoid gating) that independently control the residual strengths of the state-space interaction and the subsequent depth-wise convolutional refinement, respectively. Accordingly, $F_{\mathrm{ssm}}\left(X_{0}^{\prime }\right)=X^{\left(3\right)}$, constituting a unified feature transformation process co-driven by local structural modeling and state space interaction.

#### 3.4.3. Gated Nonlinear Enhancement Mechanism

**Figure 5:**
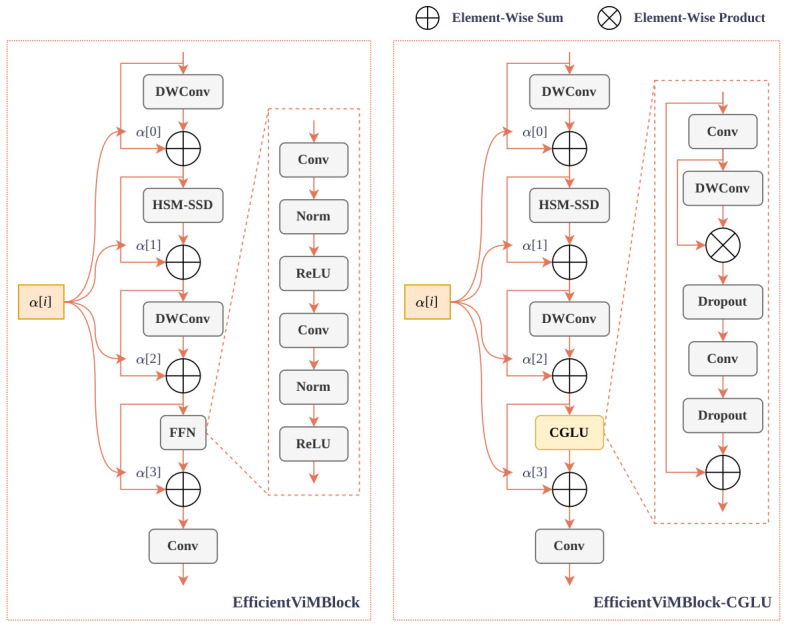
Structural comparison of the original EfficientViMBlock and the proposed EfficientViMBlock-CGLU. α[i] denotes learnable channel-wise scaling factors corresponding to different modulation paths.

After state-space-driven feature interaction, the features possess cross-position dependency propagation capability but still rely primarily on linear modeling, which cannot fully characterize complex nonlinear patterns. To address this, a Convolutional Gated Linear Unit (CGLU) is introduced as a gated nonlinear enhancement mechanism within the feature mixing mapping $F_{\mathrm{mix}}\left(\cdot \right)$ (Equation (14)). As shown in Figure 5, this replaces the standard feed-forward network (FFN) in the original EfficientViMBlock, constructing a new EfficientViMBlock-CGLU that jointly couples state space modeling with gated nonlinear feature selection.

Given feature $X^{\left(3\right)}$ after state space interaction, a linear mapping is first applied in the channel dimension, then split into a feature branch and a gate branch:

(19) $$\left[X_{f},X_{g}\right]=C_{\mathrm{expand}}\left(X^{\left(3\right)}\right).$$

Here $C_{\mathrm{expand}}\left(\cdot \right)$ denotes a pointwise (1×1) convolution that expands the channel dimension, followed by an equal split along the channel axis into a feature branch $X_{f}$ and a gate branch $X_{g}$ (the expansion ratio follows mlp_ratio=4.0).

Local spatial modeling is applied to the feature branch, which is then modulated element-wise by the gate branch:

(20) $$X^{\prime }=C_{\mathrm{dw}}\left(X_{f}\right)⊙X_{g},$$

where $C_{\mathrm{dw}}\left(\cdot \right)$ denotes a depth-wise convolution mapping. A linear mapping is then applied to restore the original channel dimension, combined with a residual path for the final update:

(21) $$X^{\left(4\right)}=X^{\left(3\right)}+C_{\mathrm{proj}}\left(X^{\prime }\right).$$

where $C_{\mathrm{proj}}\left(\cdot \right)$ denotes a pointwise (1×1) convolution that projects the gated features back to the original channel dimension before the residual addition.

This gated nonlinear enhancement mechanism is not an independently added module but forms an integral part of the feature mixing mapping $F_{\mathrm{mix}}\left(\cdot \right)$ together with state space modeling. As shown in Figure 5, the original EfficientViMBlock uses a standard feed-forward network for per-channel nonlinear transformation, while this work replaces it with a Convolutional Gated Linear Unit (CGLU) to construct EfficientViMBlock-CGLU. This modification extends the feature transformation process from a single per-channel nonlinear mapping to a “feature modeling–gate selection” jointly driven nonlinear modeling process, significantly improving the flexibility and discriminability of feature representations. From a more fundamental perspective, this design introduces an explicit gated nonlinear modulation mechanism into the state space modeling framework, extending feature modeling from a purely linear dependency propagation process to a unified modeling paradigm co-driven by dependency propagation and adaptive feature selection.

It is worth noting that CGLU differs from existing gated mechanisms in two key aspects. First, unlike standard GLU [40] and its variants such as SwiGLU and GEGLU, which perform purely channel-wise gating without explicit spatial modeling, CGLU incorporates a depth-wise convolution ($C_{\mathrm{dw}}$) in the feature branch (Equation (20)) to capture local spatial structure before gating. This spatial awareness is particularly important for dense small-object detection, where adjacent objects require fine-grained spatial discrimination. Second, unlike RemDet’s GatedFFN [28], where gated enhancement and long-range dependency modeling operate in separate, independent stages, CGLU is structurally coupled with state space modeling within the same feature mixing mapping $F_{\mathrm{mix}}\left(\cdot \right)$, enabling the gate branch to modulate features that already carry cross-position dependency information from the preceding SSM interaction (Equations (16)–(18)).

## 4. Experiments

### 4.1. Datasets and Experimental Settings

To evaluate the detection performance of the proposed method, experiments are primarily conducted on the VisDrone2019 dataset, and cross-dataset evaluation is conducted on the DIOR and UAVVaste datasets. These three datasets differ significantly in scene type, number of categories, and object scale distribution, enabling evaluation of the model’s adaptability and robustness from different perspectives.

**VisDrone2019** [2,4] is a UAV-scenario object detection dataset containing 10 categories, with 6471, 548, and 1610 images in the training, validation, and test sets, respectively. The dataset is characterized by complex backgrounds, dense objects, and high proportions of small objects, and is used as the primary experimental dataset.

**DIOR** [21] is a large-scale remote sensing object detection dataset containing 20 categories, with 16,424, 2346, and 4693 images in the training, validation, and test sets, respectively. Compared to VisDrone2019, its scene types and category compositions are more diverse, enabling evaluation of the model’s detection performance in complex remote sensing scenarios.

**UAVVaste** [22] is a UAV aerial garbage detection dataset containing only 1 category, with 540, 116, and 116 images in the training, validation, and test sets, respectively. Although relatively small in scale, its task is clearly defined and can be used to validate the model’s detection effectiveness in specific application scenarios.

**Figure 6:**
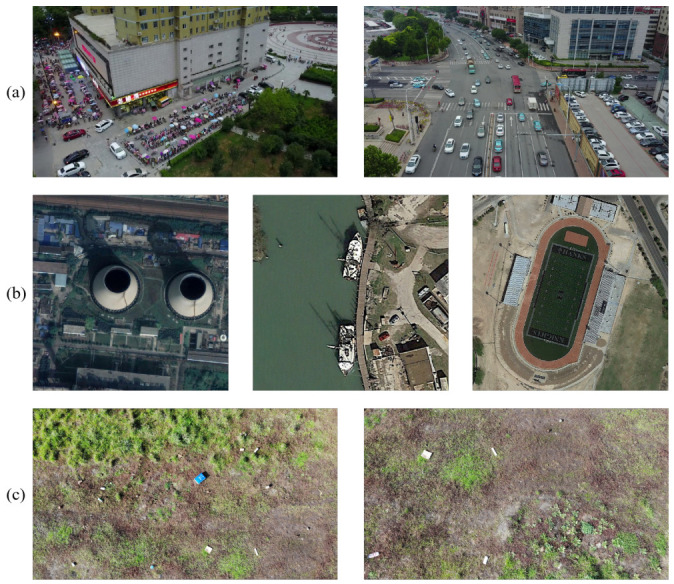
Representative samples from the three datasets. (**a**) VisDrone2019: dense urban UAV scenes; (**b**) DIOR: diverse optical remote sensing scenes; (**c**) UAVVaste: sparse aerial garbage detection.

As shown in Figure 6, the three datasets differ significantly in scene content and object presentation. VisDrone2019 is primarily composed of urban scenes from a UAV perspective with dense object distribution and complex backgrounds; DIOR covers diverse remote sensing scenes such as harbors, storage tanks, and stadiums with more complex categories and spatial structures; UAVVaste features relatively simple scenes but with extremely small and sparsely distributed objects.

**Figure 7:**
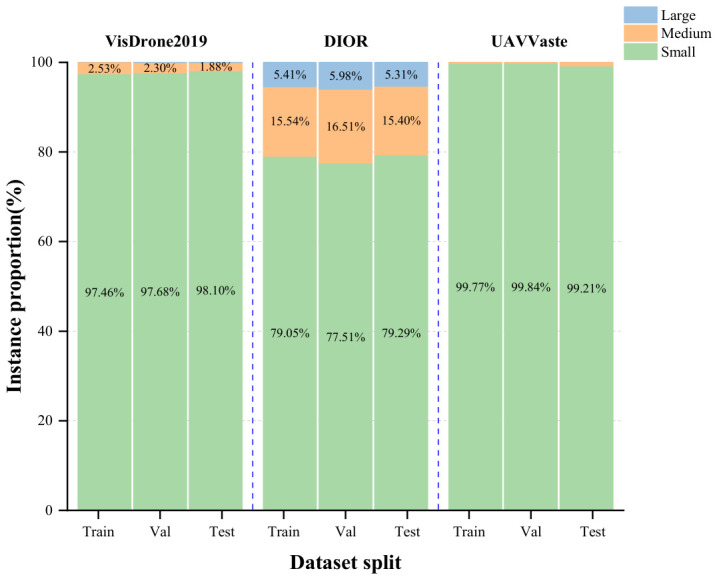
Instance scale distribution statistics across the three datasets.

To further analyze the object scale distribution of each dataset, normalized area $a=\frac{w\times h}{W\times H}$ is adopted to partition instance sizes, where *w* and *h* denote the width and height of the bounding box, and *W* and *H* denote the image width and height. Small objects satisfy a<0.01, medium objects satisfy 0.01≤a<0.1, and large objects satisfy a≥0.1. Figure 7 presents the instance scale ratio statistics for each dataset. The three datasets are all dominated by small objects: the small-object proportion in VisDrone2019 is approximately 97–98%; in DIOR approximately 78–79% with a certain proportion of medium and large objects; and in UAVVaste almost entirely small objects with a proportion exceeding 99%.

All experiments are conducted on an NVIDIA GeForce RTX 4090 D GPU, implemented based on PyTorch 2.3.0 and CUDA 12.1. The DEIM (D-FINE-S) baseline and all PFE-Det variants are trained from scratch with random initialization (backbone pretrained weights disabled; reproducibility seed set to 0). Following the DEIM training recipe and the composite objective in Equation (2), we use AdamW (learning rate $4\times 10^{-4}$ for the head/encoder/decoder and $2\times 10^{-4}$ for the backbone; weight decay $1\times 10^{-4}$; β=(0.9,0.999)) with a flat-cosine learning-rate schedule (2000-iteration warmup; lr_gamma =0.5), EMA (decay =0.9999), and a total batch size of 4. The input resolution is uniformly set to 640×640. On VisDrone2019 and UAVVaste, models are trained for 300 epochs with strong augmentation disabled after epoch 288; on DIOR, training lasts 250 epochs with strong augmentation disabled after epoch 238. No early stopping is applied. On VisDrone2019, models are trained on the training split. The Stage-2 best checkpoint is selected on the validation split, and all reported detection metrics are evaluated on the test-dev split (1610 images) under COCO-style metrics. For the competing detectors, we train and evaluate each method on VisDrone2019 using its official default configuration, without additional VisDrone-specific hyperparameter tuning; RemDet-S [28] and FRFDet-T/S/M [41] are included as recent lightweight UAV detectors under their respective official default configurations.

### 4.2. Evaluation Metrics

To comprehensively evaluate the model’s detection performance and computational overhead, assessment is conducted from two aspects: model complexity and detection accuracy. In terms of model complexity, the number of parameters (Params) and computational load (GFLOPs) are adopted as indicators. For runtime efficiency, we further report checkpoint size on disk, end-to-end inference latency (forward pass plus post-processing), and frames per second (FPS) under a unified protocol: NVIDIA GeForce RTX 4090 D, batch size 1, and 640×640 inputs on the VisDrone2019 test-dev split. Checkpoint size serves as a storage footprint indicator. In terms of detection performance, COCO standard evaluation metrics are used, including AP, ${\mathrm{AP}}_{50}$, ${\mathrm{AP}}_{s}$, ${\mathrm{AP}}_{m}$, and ${\mathrm{AP}}_{l}$. AP denotes average precision over IoU thresholds from 0.50 to 0.95 (step size 0.05); ${\mathrm{AP}}_{50}$ corresponds to detection performance at an IoU threshold of 0.50; ${\mathrm{AP}}_{s}$, ${\mathrm{AP}}_{m}$, and ${\mathrm{AP}}_{l}$ evaluate the model’s detection capability on objects of different scales. ${\mathrm{AP}}_{75}$ is additionally reported in the ablation experiments to provide finer localization precision analysis.

### 4.3. Ablation Study

To validate the effectiveness of each core component and their synergistic mechanism in the proposed method, rigorous ablation experiments are conducted on the VisDrone2019 test-dev split with DEIM-D-FINE-S as the baseline (Baseline). Quantitative results are shown in Table 1.

As shown in Table 1, the independent introduction of each module yields varying degrees of detection performance improvement, validating the soundness of the corresponding designs.

**FAENet alone:** AP improves from 0.203 to 0.211, and ${\mathrm{AP}}_{s}$ from 0.118 to 0.125. This result confirms that FAENet’s high-low frequency decoupling mechanism based on the Laplacian pyramid can alleviate the smoothing effect of early convolutional layers on fine-grained information of extremely small objects. By separating and adaptively enhancing low-frequency structure and high-frequency details, the network’s capacity for fine-grained feature retention is enhanced. Meanwhile, this preprocessing module adds only 0.04 M parameters, preserving favorable architectural compactness.

**MFAM alone:** Overall AP improves to 0.218, and medium-object precision ${\mathrm{AP}}_{m}$ reaches 0.312. Unlike traditional single convolutional paths where fine-grained structures are continuously overwritten, MFAM reorganizes the single path into independent structure-retaining and progressive enhancement paths through multi-path feature decomposition, effectively preserving local structural information and transmitting it stably to deeper layers. This ensures that context at different scales can be precisely aligned in a unified feature space, enhancing the model’s adaptability to drastic scale variation in UAVscenarios.

**MG-SSM Block alone:** AP improves to 0.210, while computational load (GFLOPs) decreases from the baseline’s 24.86 to 23.64. This reduction stems from its state-space-driven feature interaction structure, which maps 2D spatial features to 1D sequences and achieves global information interaction across positions through dynamic propagation of hidden states. Combined with CGLU for adaptive nonlinear feature selection, the block extracts long-range dependencies efficiently while reducing overall floating-point operations.

Beyond individual contributions, the interaction patterns among modules reveal critical dependencies within the progressive feature evolution framework.

**FAENet+MFAM:** As shown in Table 1, +FAENet+MFAM attains the same overall AP (0.218) as +MFAM alone. A scale-wise decomposition shows that the two configurations are not equivalent: ${\mathrm{AP}}_{s}$ increases from 0.129 to 0.132, whereas ${\mathrm{AP}}_{l}$ decreases from 0.427 to 0.404, with slight declines in ${\mathrm{AP}}_{50}$ (0.380 → 0.378) and ${\mathrm{AP}}_{75}$ (0.220 → 0.218). The unchanged aggregate AP therefore arises from an offset between a small-object gain and a large-object loss. Two complementary factors help explain this redistribution. First, MFAM already supplies multi-path structural preservation and hierarchical receptive-field coverage; once these mechanisms are present, medium- and large-object discrimination is largely saturated, so front-end frequency enhancement yields only limited additional benefit to the COCO-averaged AP. Second, without the encoder-level gated selection of the MG-SSM Block, the high-frequency components restored by FAENet are not selectively purified: they remain beneficial for extremely small objects with sparse pixel support, but can introduce texture-level interference over large object regions and slightly degrade localization quality. Overall, the effect of FAENet on top of MFAM is scale-selective and becomes more fully expressed once the subsequent MG-SSM stage completes the progressive evolution pathway, as indicated by the full model (AP 0.225, ${\mathrm{AP}}_{s}$ 0.134, ${\mathrm{AP}}_{l}$ 0.439).

**Combination ablation study (+FAENet+MG-SSM):** When FAENet and the MG-SSM Block are integrated simultaneously without MFAM (+FAENet+MG-SSM), AP drops to 0.196, falling below the baseline (0.203). The degradation spans multiple scales: ${\mathrm{AP}}_{s}$, ${\mathrm{AP}}_{m}$, and ${\mathrm{AP}}_{l}$ all decline relative to the baseline (0.111/0.277/0.397 vs. 0.118/0.289/0.412). From an architectural perspective, FAENet and MG-SSM operate at opposite ends of the network and target different defects—input-level frequency restoration versus encoder-level global sequence interaction. Without MFAM as an intermediate multi-path reorganization stage, FAENet-enriched high-frequency responses must traverse a conventional single-path backbone that neither separates structure-retaining from progressive-enhancement streams nor aligns multi-scale spatial contexts. The encoder therefore receives a mixture of weak object cues and spatially correlated background texture. Because selective state space models propagate information across the full sequence with a global receptive field, residual high-frequency background components are broadcast rather than suppressed, contaminating task-relevant representations and impairing both classification and localization. From an information-bottleneck viewpoint [12], FAENet increases the fine-grained information available at the network entrance; yet without MFAM as a structured bottleneck that filters and reorganizes this information, the subsequent global mixer cannot form a compact task-relevant representation and instead amplifies local noise. Consistent with this interpretation, +FAENet+MG-SSM underperforms the baseline as well as +MG-SSM alone (AP 0.210) and +FAENet alone (AP 0.211), whereas +MFAM+MG-SSM recovers AP to 0.222, indicating that MFAM provides a critical context-transition stage in the feature flow path.

**Full model:** Crucially, once MFAM is reintroduced to bridge FAENet and MG-SSM Block, the performance degradation is not only resolved but surpassed. When all three modules are integrated, the model achieves optimal detection performance with an overall AP of 0.225 and ${\mathrm{AP}}_{s}$ of 0.134. Notably, the full model’s computational overhead (36.91 GFLOPs) is slightly lower than that of +FAENet+MFAM (38.07 GFLOPs), despite incorporating the additional MG-SSM Block. This is consistent with the observation that the standalone +MG-SSM configuration (23.64 GFLOPs) consumes fewer floating-point operations than the baseline (24.86 GFLOPs): the MG-SSM Block restructures the encoder’s feature interaction mechanism using SSM-based modeling at linear complexity, which introduces a net GFLOPs reduction in the encoder stage. When integrated into the full model, this reduction partially offsets the overhead introduced by FAENet and MFAM. Although the overall computational overhead increases compared to the baseline, considering the 13.5% relative improvement in ${\mathrm{AP}}_{s}$, this increase in computational cost is acceptable in specific application scenarios with extremely high requirements for small-object recall (e.g., offline dense inspection from high altitude).

The preceding quantitative analysis validates the effectiveness of each module and their synergy through numerical metrics. To further verify the underlying mechanisms, we present qualitative visualizations that examine feature representation, spatial localization, and complex scene adaptability through feature maps, heat maps, and final detectionresults.

**Figure 8:**
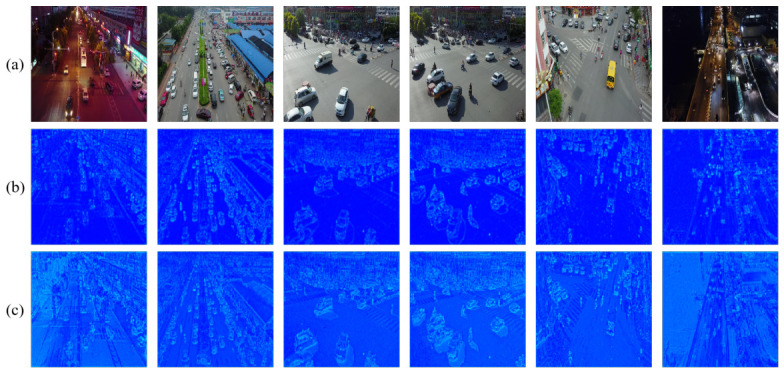
Shallow-layer feature map comparison. (**a**) Input image; (**b**) baseline model feature response; (**c**) PFE-Det feature response.

Figure 8 shows the activation states of the model in the shallow feature extraction stage. The feature maps of the baseline model exhibit predominantly attenuated and diffuse activation patterns. Due to the smoothing effect of conventional convolution operations, the high-frequency edge information of images suffers severe attenuation, making it difficult to clearly distinguish the contours of densely distributed small objects such as distant vehicles and pedestrians. In contrast, PFE-Det’s feature maps exhibit rich spatial textures and structural details, with object entities showing bright and sharp edge responses forming strong feature contrast with the surrounding background.

**Figure 9:**
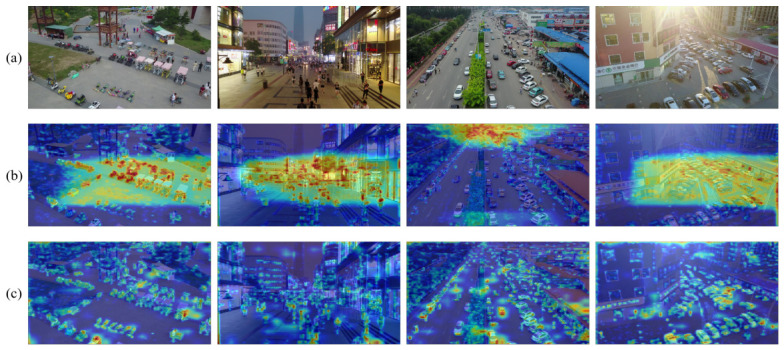
KPCA_CAM heat map visualization. (**a**) Input image; (**b**) baseline model; (**c**) PFE-Det. PFE-Det exhibits focused point-like activations on individual objects with suppressed backgroundinterference.

Figure 9 further examines the deep-layer feature representations through KPCA_CAM (Kernel PCA-based Class Activation Mapping) to visualize the nonlinear responses of features. KPCA_CAM is a gradient-free class activation mapping method that applies Kernel PCA to the activation tensors of specified target layers; in this work, a sigmoid kernel is used with the default bandwidth parameter $\gamma =1/n_{\mathrm{features}}$. The baseline model’s heat distribution shows a significant “contiguous blob” phenomenon when facing dense non-motorized vehicles, commercial street crowds, and parking lot traffic, indicating that the model cannot effectively separate densely arranged adjacent entities due to degradation of spatial structure information during continuous transformations. After introducing the improved architecture, the heat map’s spatial distribution undergoes a marked transformation: the large-area contiguous responses are effectively broken up, replaced by highly focused discrete point-like activation regions precisely anchored on individual vehicle and pedestrian instances, with activation of irrelevant backgrounds significantly suppressed.

**Figure 10:**
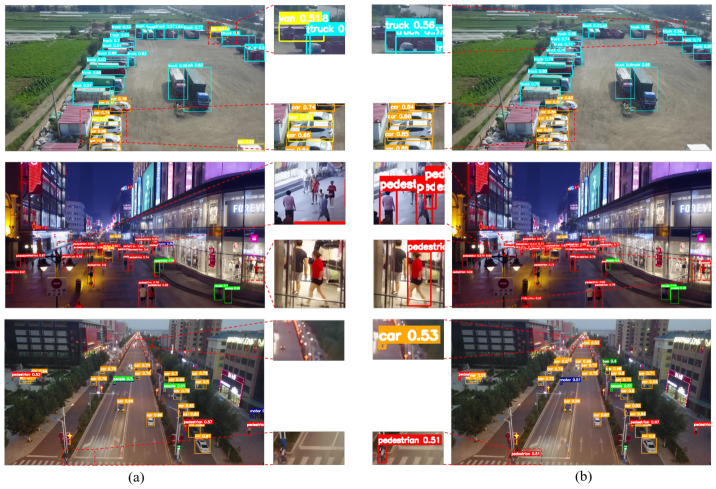
Detection result comparison. (**a**) Baseline model; (**b**) PFE-Det. PFE-Det achieves better recall of difficult small objects and eliminates inter-class confusion in dense scenes.

Finally, Figure 10 presents the final detection result comparison in actual complex UAV-perspective scenarios. Two improvements are observed: first, under extremely low illumination and long shadow scenes, PFE-Det demonstrates superior small-object recall capability; second, in complex scenes with extremely dense objects and high inter-class feature similarity, PFE-Det significantly suppresses false detections and misclassifications caused by local feature confusion.

### 4.4. Comparison Experiments

**Figure 11:**
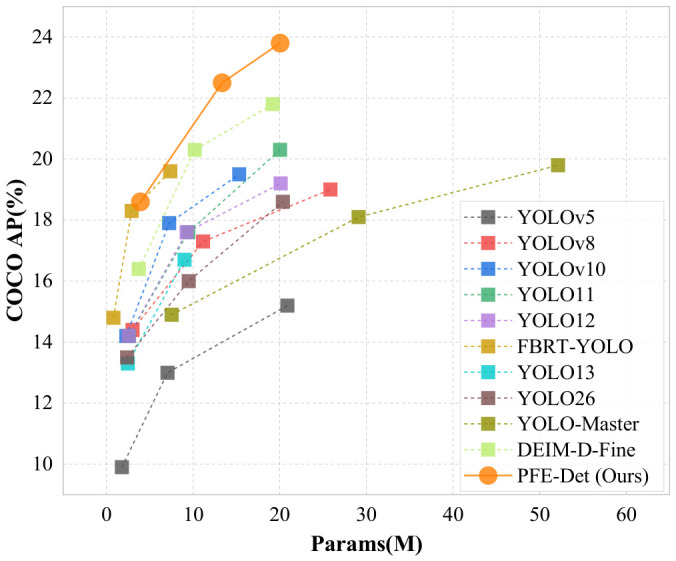
Accuracy (AP) vs. number of parameters (M) comparison of scalable detector families on VisDrone2019 test-dev. The proposed PFE-Det series occupies a favorable region in the upper-left trade-off space among the reported configurations.

To comprehensively validate the comparative performance of the proposed method, detailed experiments are conducted on the VisDrone2019 test-dev split, comparing the proposed model (PFE-Det-N, PFE-Det-S, PFE-Det-M) with representative object detection algorithms. Comparison models cover classical two-stage networks (Faster R-CNN [6], Cascade R-CNN [42]), mainstream single-stage and anchor-free networks (RetinaNet [43], TOOD [44], GFL [45], RTMDet [46], YOLOX [47], RT-DETR [9]), the latest YOLO series variants (YOLOv5, YOLOv8, YOLOv10, and YOLO11–YOLO13 [48], YOLOE-11m [49], YOLO26 [50], YOLO-Master [51], and FBRT-YOLO [52]), and recent lightweight UAV detectors RemDet-S [28] and FRFDet-T/S/M [41]. Quantitative performance evaluation metrics are detailed in Table 2, and the accuracy–parameters tradeoff is shown in Figure 11. In this table, PFE-Det and the DEIM baselines follow the from-scratch DEIM training protocol described above, whereas the remaining detectors are trained with their respective official default settings on the same VisDrone2019 splits and evaluated under the same COCO-style metrics on test-dev; the comparison is therefore a non-uniform reference under each method’s official default configuration.

In resource-constrained UAV edge computing platforms, the tradeoff between model accuracy and parameter count is the core metric for evaluating the practical value of an algorithm. As shown in Figure 11, the proposed PFE-Det series models construct a favorable Pareto front in the upper-left region of the coordinate system across all scalable detector families. Specifically, PFE-Det-M achieves the highest overall detection accuracy (AP of 0.238) with a moderate parameter scale of 20.05 M. As further evidenced by Table 2, this accuracy substantially exceeds that of traditional heavy networks such as Cascade R-CNN-R50-FPN (69.29 M parameters, an AP of only 0.197) and ATSS-R50-FPN-DyHead (38.91 M, AP 0.204).

In the approximately 20 M parameter track, PFE-Det-M (20.05 M, AP 0.238) demonstrates clear performance superiority. Compared with the latest models at a comparable scale, YOLO11m (20.04 M) and YOLO12m (20.11 M) achieve an AP of only 0.203 and 0.192, respectively; compared to the baseline model DEIM-D-FINE-M (19.19 M, AP 0.218), PFE-Det-M achieves a 2.0 absolute percentage point accuracy gain with only a modest increase in computational cost (60.02 vs. 56.37 GFLOPs). At the same time, ${\mathrm{AP}}_{s}$ and ${\mathrm{AP}}_{m}$ rise from 0.129/0.307 to 0.145/0.336, while ${\mathrm{AP}}_{l}$ decreases slightly from 0.394 to 0.384 (Table 2). To examine this mild ${\mathrm{AP}}_{l}$ change, we further report class-wise ${\mathrm{AP}}_{l}$ on VisDrone2019 test-dev together with the number of large ground-truth instances under the COCO area criterion ($area\geq 96^{2}$; Table 3). Class-wise ${\mathrm{AP}}_{l}$ increases for seven categories and decreases only for people, motor, and bicycle. The aggregate ${\mathrm{AP}}_{l}$ decrease is concentrated in these three classes (−0.097/−0.130/−0.068), which together contribute only 14 large instances (0.58% of all large GTs). By contrast, ${\mathrm{AP}}_{l}$ increases for the large-object–dominant categories car, van, truck, bus, and awning-tricycle, which account for most large instances. Thus the M-scale ${\mathrm{AP}}_{l}$ dip is associated with an extremely sparse large-object subset and should be interpreted with caution. Consistently, PFE-Det-N and PFE-Det-S improve ${\mathrm{AP}}_{l}$ over their DEIM counterparts on VisDrone (0.336 → 0.368 and 0.412 → 0.439), and PFE-Det-S also improves ${\mathrm{AP}}_{l}$ on DIOR and UAVVaste (0.795 → 0.803 and 0.590 → 0.635).

In the lightweight track, PFE-Det-S (13.33 M) also achieves a high AP of 0.225, consistently surpassing YOLOv8s (11.13 M, AP 0.173) and YOLO-Master-n (7.51 M, AP 0.149). RemDet-S (12.84 M, AP 0.204) attains comparable overall accuracy to DEIM-D-FINE-S (0.203) at lower computational cost (16.31 GFLOPs). FRFDet-T (2.60 M, AP 0.161) offers a more compact UAV-oriented baseline with 9.8 GFLOPs, but trails PFE-Det-N (3.92 M, AP 0.186; ${\mathrm{AP}}_{s}$ 0.105 vs. 0.073) under the same test-dev COCO protocol. FRFDet-S (9.33 M, AP 0.190; ${\mathrm{AP}}_{s}$ 0.091) remains below PFE-Det-S (13.33 M, AP 0.225; ${\mathrm{AP}}_{s}$ 0.134), and FRFDet-M (17.42 M, AP 0.213; ${\mathrm{AP}}_{s}$ 0.110) remains below PFE-Det-M (20.05 M, AP 0.238; ${\mathrm{AP}}_{s}$ 0.145) despite higher GFLOPs (98.0 vs. 60.02).

Regarding the critical challenge of extremely small object detection in UAV aerial scenarios, PFE-Det achieves substantial gains in ${\mathrm{AP}}_{s}$. As shown in Table 2, current mainstream advanced models encounter severe performance bottlenecks when facing small objects (e.g., TOOD-R50 ${\mathrm{AP}}_{s}$ 0.102, YOLO-Master-m ${\mathrm{AP}}_{s}$ 0.100, Cascade R-CNN ${\mathrm{AP}}_{s}$ 0.099). However, the lightest PFE-Det-N (3.92 M) already achieves an ${\mathrm{AP}}_{s}$ of 0.105, surpassing the majority of medium-to-large networks; and the ${\mathrm{AP}}_{s}$ of PFE-Det-M reaches 0.145, setting a strong reference among the compared methods for small-object detection.

From the computational efficiency perspective, the proposed models achieve a favorable accuracy–GFLOPs tradeoff compared to methods at similar accuracy levels. PFE-Det-S (36.91 GFLOPs) attains an AP of 0.225, substantially exceeding YOLOv5m (48.0 GFLOPs, AP 0.152) and YOLOv10m (58.9 GFLOPs, AP 0.195), which require 30–60% more computation yet deliver considerably lower accuracy. Among methods in the 60–70 GFLOPs range, PFE-Det-M (60.02 GFLOPs, AP 0.238) surpasses YOLO11m (67.7 GFLOPs, AP 0.203), YOLO12m (67.2 GFLOPs, AP 0.192), and YOLO26m (67.9 GFLOPs, AP 0.186) while consuming approximately 11% fewer floating-point operations. Compared to traditional heavy architectures such as Faster R-CNN (208 GFLOPs, AP 0.194) and GFL (206 GFLOPs, AP 0.193), PFE-Det-S achieves markedly higher accuracy at less than one-fifth of the computational cost.

Table 4 further compares the DEIM baselines and PFE-Det variants under the unified runtime protocol above. Relative to the matched DEIM counterpart, PFE-Det-N/S/M increase end-to-end latency from 9.58/10.56/14.83 ms to 12.55/16.29/18.23 ms and reduce FPS from 104.38/94.65/67.41 to 79.69/61.37/54.85, while checkpoint size grows modestly (14.5 → 15.2 MB, 39.2 → 51.3 MB, and 73.7 → 77.0 MB). The S-scale overhead is the most pronounced because progressive feature evolution expands multi-path and multi-scale modeling in the backbone–encoder pathway; nevertheless, PFE-Det-S still sustains real-time throughput above 60 FPS on an RTX 4090 D, and the corresponding AP/${\mathrm{AP}}_{s}$ gains (0.203 → 0.225 and 0.118 → 0.134) indicate a favorable accuracy–latency tradeoff for offline or ground-station processing. The present desktop-GPU latency/FPS figures therefore serve as a high-end reference; watt-level energy metering and onboard measurements on embedded UAV platforms (e.g., Jetson-class devices) are left for future work, and edge deployment remains contingent on complementary compression and acceleration (Section 5).

### 4.5. Cross-Dataset Validation

To evaluate cross-dataset applicability, we conduct independent training-and-testing experiments on the DIOR large-scale remote sensing dataset and the UAVVaste UAV garbage detection dataset. Specifically, both PFE-Det-S and the baseline DEIM-D-FINE-S are trained on each target dataset using the same DEIM training recipe without dataset-specific hyperparameter tuning (optimizer, augmentation policy, and learning-rate schedule); training epochs follow the dataset configuration (250 for DIOR and 300 for UAVVaste). All results are shown in Table 5 and Table 6.

The DIOR dataset covers 20 complex remote sensing categories, with a significantly higher proportion of medium and large objects compared to VisDrone (medium and large objects combined account for approximately 21% of instances in DIOR, versus only ∼2–3% in VisDrone). As shown in Table 5, without targeted hyperparameter tuning, PFE-Det-S’s overall AP on DIOR improves from 0.650 to 0.657. Notably, the model demonstrates favorable adaptability on medium objects (${\mathrm{AP}}_{m}$ from 0.508 to 0.517) and large objects (${\mathrm{AP}}_{l}$ from 0.795 to 0.803). A slight decrease in ${\mathrm{AP}}_{s}$ of 0.006 (0.311 to 0.305) occurs; under unfamiliar wide-area remote sensing noise such as cloud layers and terrain textures, the MG-SSM module’s global gating mechanism tends to apply a stricter feature purification strategy. The increase in ${\mathrm{AP}}_{75}$ from 0.705 to 0.711 demonstrates that the model achieves an overall improvement in detection quality at the cost of a slight reduction in edge small-object recall.

**Table 5:** Cross-dataset validation results on DIOR. The better result for each metric is in **bold**.

Model	AP	${\mathrm{AP}}_{50}$	${\mathrm{AP}}_{75}$	${\mathrm{AP}}_{s}$	${\mathrm{AP}}_{m}$	${\mathrm{AP}}_{l}$	Params (M)	GFLOPs
DEIM-D-FINE-S	0.650	0.861	0.705	**0.311**	0.508	0.795	10.19	24.90
PFE-Det-S	**0.657**	**0.868**	**0.711**	0.305	**0.517**	**0.803**	13.34	36.95

The UAVVaste dataset presents distribution characteristics distinctly different from VisDrone: backgrounds are mainly natural textures (grass, beach), and targets (garbage) are extremely small (small objects exceeding 99%) and very sparsely distributed. As shown in Table 6, PFE-Det-S achieves clear performance gains on this task: overall AP increases by 2.9 percentage points (0.500 to 0.529, a relative improvement of 5.8%), and ${\mathrm{AP}}_{50}$ reaches 0.830. Notably, ${\mathrm{AP}}_{75}$, measuring high localization precision, improves by 4.6 percentage points (0.557 to 0.603). The MFAM structure-retaining path helps preserve boundary cues of sparse targets, coinciding with the gains in overall AP and ${\mathrm{AP}}_{75}$. Meanwhile, a marginal decrease of 0.006 in ${\mathrm{AP}}_{s}$ (0.259 to 0.253) is observed, consistent with the slight ${\mathrm{AP}}_{s}$ reduction on DIOR. Given that over 99% of UAVVaste instances fall within the small-object category, the substantial overall AP gain (+2.9 points) occurring alongside a minor ${\mathrm{AP}}_{s}$ dip suggests that the improvement is not driven by a change in COCO size-bin membership. Under COCO evaluation, the “small/medium/large” partition is determined by the ground-truth bounding-box area; therefore, predicted box refinement does not alter the GT-based size bin. Instead, the observed ${\mathrm{AP}}_{s}$/${\mathrm{AP}}_{m}$ differences reflect changes in detection quality for the corresponding GT size subsets (and the remaining non-small instances).

**Table 6:** Cross-dataset validation results on UAVVaste. The better result for each metric is in **bold**.

Model	AP	${\mathrm{AP}}_{50}$	${\mathrm{AP}}_{75}$	${\mathrm{AP}}_{s}$	${\mathrm{AP}}_{m}$	${\mathrm{AP}}_{l}$	Params (M)	GFLOPs
DEIM-D-FINE-S	0.500	0.810	0.557	**0.259**	0.534	0.590	10.18	24.82
PFE-Det-S	**0.529**	**0.830**	**0.603**	0.253	**0.562**	**0.635**	13.32	36.87

Combining the performance on both cross-dataset evaluations, PFE-Det shows consistent scene adaptability: overall AP improves on DIOR and UAVVaste under the shared training recipe, while ${\mathrm{AP}}_{s}$ decreases slightly on both datasets. These results indicate favorable overall detection quality across the evaluated aerial and remote sensing benchmarks, without implying zero-shot domain transfer.

## 5. Discussion

### 5.1. Core Results Review and Comparative Analysis with Existing Research

We next position PFE-Det relative to the detector families most closely related to its baseline and technical ingredients. The experimental results position the proposed framework distinctly from prior UAV detection improvements along three axes. First, compared with detection-stage compensation approaches such as TPH-YOLOv5 [8], which stacks additional prediction heads to cover different scales, the proposed method intervenes earlier and more fundamentally in the feature extraction pipeline. The 13.5% relative ${\mathrm{AP}}_{s}$ improvement achieved by PFE-Det-S exceeds the gains typically obtained by head-level modifications alone, because structural fidelity is preserved before features reach the detection head rather than being retrospectively compensated. Second, compared with lightweight UAV-oriented designs such as RemDet [28], CEASC [27], and FRFDet [41], the proposed method explicitly targets feature-level structural preservation rather than computational reduction alone. While RemDet’s GatedFFN introduces gated modeling in the feed-forward stage, its gating mechanism operates independently of long-range dependency extraction; the MG-SSM Block, by contrast, couples these two functions within a single residual stage, explaining the consistent ${\mathrm{AP}}_{s}$ advantage of PFE-Det-S over RemDet-S (0.134 vs. 0.099; Table 2) under the same VisDrone test-dev protocol. FRFDet instead emphasizes symmetric sampling and scale-aware fusion for compact UAV detectors; under the same test-dev COCO protocol, PFE-Det-N/S/M still outperform the matched FRFDet-T/S/M counterparts in overall AP and ${\mathrm{AP}}_{s}$ (Table 2), indicating that progressive structural protection along the feature pathway yields complementary gains beyond sampling- and fusion-centric redesigns. Third, compared with the information-theoretic motivation of YOLOv9 [12], which addresses information loss via Programmable Gradient Information during backpropagation, the proposed framework tackles the same underlying problem from the forward-propagation perspective through explicit frequency decoupling and multi-path structural retention, offering a complementary and architecturally distinctsolution.

Relative to recent DEIM-, RT-DETR-, and Mamba-based detectors, the difference lies mainly in where and how structural information is protected. DEIM improves dense one-to-one matching and convergence on a given feature hierarchy, but leaves the low-to-high feature evolution path that feeds the decoder largely unchanged; PFE-Det therefore retains DEIM as the detection baseline while reconstructing the preprocessing–backbone–encoder pathway that determines which structural cues remain available for matching. RT-DETR-style hybrid encoders emphasize efficient multi-scale feature interaction and query selection, yet early convolutional smoothing and single-path overwriting can still accumulate upstream of the encoder; progressive feature evolution targets these upstream defects before encoder fusion. Mamba-/SSM-based detectors provide linear-complexity global modeling, typically by replacing or augmenting a token mixer; the MG-SSM Block further couples HSM-SSD dependency extraction with CGLU-based spatially aware selection and is placed after MFAM has reorganized multi-scale structure, consistent with the ordered dependency observed in ablation. In this sense, progressive feature evolution provides a staged pathway that (i) adopts FAENet for source-level frequency restoration, (ii) designs MFAM to couple multi-path retention with hierarchical receptive-field modeling, and (iii) constructs MG-SSM for joint dependency extraction and gated selection, forming a coherent optimization chain for UAV small-object detection.

### 5.2. Scientific Implications and Mechanism Interpretation

Having established the proposed framework’s comparative position, we now examine the mechanistic basis underlying its performance characteristics. The ablation results reveal two complementary interaction patterns along the feature flow. First, the identical overall AP of +FAENet+MFAM and +MFAM (both 0.218) is explained by a scale-wise offset: FAENet improves ${\mathrm{AP}}_{s}$ while slightly reducing ${\mathrm{AP}}_{l}$ when encoder-level gated selection is absent, indicating that front-end frequency enrichment is beneficial but incomplete without the full progressive pathway. Second, the performance drop when FAENet and the MG-SSM Block are combined without MFAM (AP falling to 0.196, below the baseline) can be interpreted through the lens of information bottleneck theory [12]. FAENet enriches the input with high-frequency structural details that would otherwise be lost, but when these details propagate through a single-path backbone to the encoder without intermediate multi-scale reorganization, the state space model’s global receptive field indiscriminately propagates both signal and spatially correlated noise across the entire feature sequence. The accompanying declines in ${\mathrm{AP}}_{s}$, ${\mathrm{AP}}_{m}$, and ${\mathrm{AP}}_{l}$ further indicate a pathway-level disruption of feature quality. MFAM alleviates this issue by acting as a structured bottleneck that separates stable structural representations from progressively enhanced features before they enter the encoder, so that the MG-SSM Block receives spatially organized input amenable to efficient sequential modeling. Together, these patterns point to an ordered dependency chain in which MFAM serves as the critical intermediate stage.

The visualization results provide converging evidence for this interpretation. Shallow feature maps (Figure 8) confirm that FAENet’s frequency decoupling produces high-contrast edge responses where the baseline shows diffuse activations—a direct visual manifestation of smoothing effect mitigation. More importantly, the KPCA_CAM heat maps (Figure 9) reveal that the transition from coarse “contiguous blob” activations to focused point-like responses occurs specifically at deep layers, confirming that MFAM’s multi-path decomposition successfully prevents the feature overwriting that would otherwise accumulate across the backbone’s sequential transformations. This observation aligns with the broader finding from MetaFormer [19] that the residual framework structure, rather than any specific token mixer, drives representational quality—the MG-SSM Block leverages precisely this principle by embedding state space modeling within a two-stage residual architecture.

### 5.3. Innovation Value and Advantages

Taken together, these comparisons indicate that the value of PFE-Det lies in organizing frequency restoration, multi-path structural retention, and gated state-space interaction into a coherent progressive pathway. The mechanistic insights above explain why PFE-Det achieves strong numerical results; we now examine how these mechanisms translate into tangible detection improvements in practical scenarios. Beyond quantitative metrics, the framework’s practical value is most evident in two concrete detection scenarios revealed by the qualitative analysis (Figure 10). In extremely low-illumination and long-shadow scenes, where small objects share similar intensity profiles with the background, the frequency decoupling mechanism of FAENet preserves discriminative edge information that conventional backbones discard during early downsampling, enabling the model to recall targets that are effectively invisible to the baseline detector. In dense parking-lot and commercial-street scenes with high inter-class similarity (e.g., distinguishing non-motorized vehicles from pedestrians at comparable scales), the multi-path structural retention of MFAM prevents the spatial confusion that causes the baseline’s merged activation blobs, while the MG-SSM Block’s gated selection mechanism provides the discriminative nonlinearity needed to separate semantically similar categories. The cross-dataset results on DIOR and UAVVaste further indicate that these advantages are not dataset-specific: the architecture generalizes without hyperparameter tuning to remote sensing scenes with different category distributions and to single-category sparse detection tasks. These observations suggest that progressive feature evolution captures fundamental properties of the feature extraction process rather than dataset-specific patterns, and may serve as a useful design principle for aerial and remote sensing detection architectures.

### 5.4. Research Limitations and Future Outlook

Despite the gains observed in small-object detection and complex-scene adaptability, several limitations warrant acknowledgment.

The first concerns the pronounced inter-module structural dependency. Combination ablation experiments show that when FAENet and the MG-SSM Block are integrated simultaneously without MFAM, the model’s overall AP drops to 0.196, with concurrent declines across ${\mathrm{AP}}_{s}$, ${\mathrm{AP}}_{m}$, and ${\mathrm{AP}}_{l}$. Without MFAM for context transition and multi-scale structured reorganization, high-frequency details extracted by the front end may be amplified as local noise when they enter the back-end state space with a global receptive field. Relatedly, the unchanged overall AP of +FAENet+MFAM relative to +MFAM suggests that the benefit of FAENet remains only partially expressed until encoder-level gated selection is available. Future work may investigate the alignment and co-evolution mechanisms between multi-level structural features and the global state space from a representation learning perspective to enhance the stability of module decoupling.

Second, fully integrating the three modules increases computational overhead from the baseline’s 24.86 GFLOPs to 36.91 GFLOPs and, under the unified runtime protocol in Table 4, raises end-to-end latency from 10.56 ms to 16.29 ms for the S-scale pair (94.65 → 61.37 FPS). Although acceptable for offline high-altitude inspection with stringent small-object recall requirements, this cost constrains real-time deployment on resource-limited micro-UAV edge devices. Future work will further profile peak GPU memory, meter watt-level energy, and benchmark onboard performance on embedded UAV platforms, while developing lighter multi-scale reconstruction operators, state-space compression, and edge-oriented model compression (e.g., quantization and distillation), consistent with recent analyses of efficiency constraints in onboard UAV perception [60].

Third, large objects remain scarce on VisDrone test-dev (only 3.21% of instances under the COCO area criterion; Table 3), so ${\mathrm{AP}}_{l}$ is intrinsically more variable than ${\mathrm{AP}}_{s}$ and ${\mathrm{AP}}_{m}$. Class-wise evaluation shows that the mild PFE-Det-M ${\mathrm{AP}}_{l}$ decrease is concentrated in categories with almost no large instances; multi-seed variability analysis is left for futurework.

Fourth, key architectural settings of FAENet, MFAM, and the MG-SSM Block are fixed according to the constrained design choices and task-oriented rationales in Section 3. A systematic sensitivity sweep over alternative configurations (e.g., pyramid depth, channel-group cardinality, progressive-block depth, and MG-SSM placement) is likewise reserved for future work.

Fifth, the present evaluation follows the standard VisDrone2019, DIOR, and UAVVaste splits. Future work will further construct controlled stress tests under explicit UAV sensing degradations—such as altitude-induced scale shift, adverse weather, strong illumination change, motion blur, or camera vibration—to assess field reliability beyond cross-dataset scene and category transfer.

Sixth, the present study focuses on still-image detection under a fixed closed-set vocabulary. Extending progressive feature evolution to video object detection and open-vocabulary aerial perception—including track-by-description and broader situational awareness pipelines discussed in recent UAV detection surveys [60]—remains an important next step for continuous aerial monitoring.

Finally, beyond single-platform perception, real-world UAV intelligence increasingly involves multi-UAV collaboration and energy-efficient aerial communication. Recent work on multi-UAV networks with RIS-assisted rate-splitting transmission highlights trajectory, resource, and energy constraints that co-exist with onboard sensing [61]. Integrating progressive feature evolution with multi-UAV cooperative perception and communication-aware deployment therefore constitutes a broader research avenue toward intelligent UAVapplications.

## 6. Conclusions

This paper presents PFE-Det, a progressive feature evolution framework that systematically addresses the cascading degradation of structural information in UAV aerial small-object detection. Built upon DEIM, PFE-Det constructs a three-stage continuous evolution path—structural enhancement via FAENet, multi-scale reconstruction via MFAM, and global dependency modeling with adaptive selection via MG-SSM Block—that progressively optimizes feature representations from input to encoder.

Experimental results demonstrate that PFE-Det substantially mitigates the fundamental defects of fine-grained features being smoothed and overwritten during continuous forward propagation in traditional networks. On the VisDrone2019 test-dev split, PFE-Det-S achieves an overall AP of 0.225 and small-object precision ${\mathrm{AP}}_{s}$ of 0.134, representing a 13.5% relative improvement over the baseline. This performance gain validates the synergistic mechanism of the three modules: FAENet mitigates the smoothing effect on fine-grained information through high-low frequency decoupling; MFAM preserves local structural information and transmits it stably to deeper layers via multi-path decomposition; and MG-SSM Block achieves efficient global dependency extraction and feature purification through 1D hidden state propagation and gated nonlinear modulation. Additionally, cross-dataset validation results further confirm that the architecture retains favorable adaptability when dealing with complex remote sensing scenarios and specific UAV aerial tasks.

In summary, this work establishes progressive feature evolution as a viable paradigm for small-object detection in complex UAV visual environments, offering a principled alternative to post-hoc detection-stage compensation. Although the integrated framework incurs moderate computational overhead (36.91 vs. 24.86 GFLOPs), the substantial detection performance improvement renders it well-suited for application scenarios with stringent small-object recall requirements, such as offline high-altitude dense inspection and post-disaster search operations. The demonstrated cross-dataset evaluation results further suggest that progressive feature evolution may benefit broader aerial and remote sensing detection tasks beyond the specific datasets evaluated in this study.

## Figures and Tables

**Table 1:** Ablation study results on VisDrone2019 test-dev. ✓ indicates the module is included. The best results are in **bold**.

Model	FAENet	MFAM	MG-SSM	AP	${\mathrm{AP}}_{50}$	${\mathrm{AP}}_{75}$	${\mathrm{AP}}_{s}$	${\mathrm{AP}}_{m}$	${\mathrm{AP}}_{l}$	Params (M)	GFLOPs
Baseline				0.203	0.356	0.203	0.118	0.289	0.412	10.18	24.86
+FAENet	✓			0.211	0.367	0.212	0.125	0.301	0.401	10.22	34.21
+MFAM		✓		0.218	0.380	0.220	0.129	0.312	0.427	12.67	28.71
+MG-SSM			✓	0.210	0.366	0.210	0.122	0.302	0.388	10.81	23.64
+FAENet+MFAM	✓	✓		0.218	0.378	0.218	0.132	0.310	0.404	12.71	38.07
+FAENet+MG-SSM	✓		✓	0.196	0.342	0.195	0.111	0.277	0.397	10.84	32.99
+MFAM+MG-SSM		✓	✓	0.222	0.385	0.223	0.129	0.318	0.430	13.30	27.55
**Full (PFE-Det-S)**	✓	✓	✓	**0.225**	**0.390**	**0.224**	**0.134**	**0.322**	**0.439**	13.33	36.91

**Table 2:** Comparison with state-of-the-art methods on VisDrone2019 test-dev. The best results are in **bold**. Italicized row labels denote detector category groupings.

Method	AP	${\mathrm{AP}}_{50}$	${\mathrm{AP}}_{s}$	${\mathrm{AP}}_{m}$	${\mathrm{AP}}_{l}$	Params (M)	GFLOPs
*Two-stage detectors*							
Faster R-CNN [6]	0.194	0.329	0.095	0.309	0.429	41.39	208
Cascade R-CNN-R50-FPN [42]	0.197	0.326	0.099	0.309	0.406	69.29	236
*Single-stage and anchor-free detectors*							
RetinaNet-R50-FPN [43]	0.164	0.276	0.060	0.274	0.427	36.52	210
GFL [45]	0.193	0.321	0.094	0.300	0.409	32.28	206
ATSS-R50-FPN-DyHead [53,54]	0.204	0.338	0.100	0.317	**0.485**	38.91	110
TOOD-R50 [44]	0.204	0.339	0.102	0.317	0.403	32.04	199
RTMDet-Tiny [46]	0.184	0.312	0.077	0.288	0.445	4.88	8.03
YOLOX-Tiny [47]	0.148	0.278	0.076	0.221	0.278	5.04	7.58
RT-DETR-R18 [9]	0.208	0.363	0.113	0.305	0.413	19.89	57
*YOLO series*							
YOLOv5n [55]	0.099	0.205	0.046	0.154	0.231	1.77	4.2
YOLOv5s [55]	0.130	0.257	0.062	0.201	0.259	7.04	15.8
YOLOv5m [55]	0.152	0.288	0.073	0.233	0.306	20.89	48.0
YOLOv8n [56]	0.144	0.259	0.059	0.225	0.339	3.00	8.1
YOLOv8s [56]	0.173	0.307	0.078	0.269	0.372	11.13	28.5
YOLOv8m [56]	0.190	0.332	0.090	0.294	0.417	25.85	78.7
YOLOv10n [57]	0.142	0.261	0.063	0.224	0.292	2.28	6.5
YOLOv10s [57]	0.179	0.323	0.086	0.278	0.361	7.22	21.4
YOLOv10m [57]	0.195	0.345	0.097	0.300	0.414	15.32	58.9
YOLO11n [58]	0.142	0.258	0.058	0.225	0.316	2.59	6.3
YOLO11s [58]	0.176	0.313	0.080	0.272	0.364	9.42	21.3
YOLO11m [58]	0.203	0.350	0.098	0.312	0.413	20.04	67.7
YOLO12n [59]	0.142	0.259	0.057	0.224	0.346	2.56	6.3
YOLO12s [59]	0.176	0.312	0.081	0.274	0.356	9.23	21.2
YOLO12m [59]	0.192	0.336	0.094	0.298	0.386	20.11	67.2
YOLO13n [48]	0.133	0.244	0.055	0.210	0.317	2.45	6.2
YOLO13s [48]	0.167	0.297	0.077	0.258	0.387	9.00	20.1
YOLOE-11m [49]	0.195	0.339	0.092	0.301	0.427	20.04	67.7
YOLO26n [50]	0.135	0.249	0.063	0.203	0.291	2.38	5.2
YOLO26s [50]	0.160	0.294	0.082	0.240	0.362	9.47	20.5
YOLO26m [50]	0.186	0.332	0.096	0.281	0.361	20.36	67.9
FBRT-YOLO-N [52]	0.148	0.265	0.062	0.234	0.323	0.80	6.7
FBRT-YOLO-S [52]	0.183	0.323	0.085	0.283	0.425	2.90	22.9
FBRT-YOLO-M [52]	0.196	0.344	0.094	0.309	0.421	7.36	58.7
YOLO-Master-n [51]	0.149	0.269	0.064	0.232	0.359	7.51	9.6
YOLO-Master-s [51]	0.181	0.318	0.085	0.281	0.359	29.10	34.3
YOLO-Master-m [51]	0.198	0.344	0.100	0.306	0.408	52.12	116.4
*Lightweight UAV detectors*							
RemDet-S [28]	0.204	0.347	0.099	0.313	0.423	12.84	16.31
FRFDet-T [41]	0.161	0.287	0.073	0.252	0.333	2.60	9.8
FRFDet-S [41]	0.190	0.333	0.091	0.297	0.364	9.33	32.7
FRFDet-M [41]	0.213	0.369	0.110	0.327	0.404	17.42	98.0
*DEIM baselines*							
DEIM-D-FINE-N [10,11]	0.164	0.295	0.089	0.238	0.336	3.73	7.12
DEIM-D-FINE-S [10,11]	0.203	0.356	0.118	0.289	0.412	10.18	24.86
DEIM-D-FINE-M [10,11]	0.218	0.378	0.129	0.307	0.394	19.19	56.37
*Proposed method*							
PFE-Det-N	0.186	0.332	0.105	0.269	0.368	3.92	18.35
PFE-Det-S	0.225	0.390	0.134	0.322	0.439	13.33	36.91
PFE-Det-M	**0.238**	**0.412**	**0.145**	**0.336**	0.384	20.05	60.02

**Table 3:** Class-wise ${\mathrm{AP}}_{l}$ of DEIM-D-FINE-M and PFE-Det-M on VisDrone2019 test-dev, with large ground-truth counts under the COCO criterion ($area\geq 96^{2}$). “# Large” denotes the number of large ground-truth instances. Δ${\mathrm{AP}}_{l}$ = PFE-Det-M − DEIM-D-FINE-M.

Category	# Large	Share of Large	${\mathrm{AP}}_{l}$ (DEIM-M)	${\mathrm{AP}}_{l}$ (PFE-Det-M)	Δ ${\mathrm{AP}}_{l}$
pedestrian	92	3.82%	0.536	0.546	+0.010
people	3	0.12%	0.143	0.046	−0.097
bicycle	5	0.21%	0.192	0.124	−0.068
car	1110	46.08%	0.780	0.790	+0.010
van	190	7.89%	0.479	0.502	+0.023
truck	437	18.14%	0.464	0.526	+0.062
tricycle	18	0.75%	0.300	0.302	+0.002
awning-tricycle	25	1.04%	0.219	0.291	+0.072
bus	523	21.71%	0.631	0.642	+0.011
motor	6	0.25%	0.197	0.067	−0.130
all/total	2409	100%	0.394	0.384	−0.010

**Table 4:** Runtime efficiency comparison between DEIM baselines and PFE-Det variants on VisDrone2019 test-dev (RTX 4090 D; batch size 1; 640×640). “Checkpoint size” denotes the saved weight file size on disk. Latency is the sum of forward inference and post-processing; FPS includes post-processing.

Model	Params (M)	Checkpoint Size (MB)	GFLOPs	Latency (ms)	FPS
DEIM-D-FINE-N	3.73	14.5	7.12	9.58	104.38
PFE-Det-N	3.92	15.2	18.35	12.55	79.69
DEIM-D-FINE-S	10.18	39.2	24.86	10.56	94.65
PFE-Det-S	13.33	51.3	36.91	16.29	61.37
DEIM-D-FINE-M	19.19	73.7	56.37	14.83	67.41
PFE-Det-M	20.05	77.0	60.02	18.23	54.85

## Data Availability

Publicly available datasets were analyzed in this study. The VisDrone2019 dataset is available at https://github.com/VisDrone/VisDrone-Dataset (accessed on 30 May 2026); the DIOR dataset is available at https://opendatalab.com/OpenDataLab/DIOR (accessed on 30 May 2026); and the UAVVaste dataset is available at https://github.com/UAVVaste/UAVVaste (accessed on 30 May 2026).

## Abbreviations

The following abbreviations are used in this manuscript:

PFE-Det	Progressive Feature Evolution Detector
UAV	Unmanned Aerial Vehicle
AP	Average Precision
FAENet	Feature Adaptive Enhancement Network
MFAM	Multi-Receptive-Field Adaptive Fusion Module
MG-SSM	Multi-Path Gated State Space Modeling
SSM	State Space Model
HSM-SSD	Hidden State Mixer-Based State Space Duality
CGLU	Convolutional Gated Linear Unit
RFEM	Receptive Field Fusion Enhancement Module
ATEM	Affine Transformation Enhancement Module
LAM	Lightweight Attention Module
LWGA	Lightweight Group Attention
SCDown	Lightweight Separable-Convolution Downsampling Operator
RepNCSPELAN4	Multi-Branch CSP-ELAN-Style Cross-Scale Fusion Block
DFINETransformer	D-FINE Detection Transformer Decoder
FPN	Feature Pyramid Network
GLU	Gated Linear Unit
CNN	Convolutional Neural Network
NMS	Non-Maximum Suppression
KPCA	Kernel Principal Component Analysis
CAM	Class Activation Map
